# Rethinking the Plant Economics Spectrum for Annuals: A Multi-Species Study

**DOI:** 10.3389/fpls.2021.640862

**Published:** 2021-03-26

**Authors:** Susanne Kurze, Bettina M. J. Engelbrecht, Mark C. Bilton, Katja Tielbörger, Leonor Álvarez-Cansino

**Affiliations:** ^1^Functional and Tropical Plant Ecology, Bayreuth Centre of Ecology and Environmental Research (BayCEER), University of Bayreuth, Bayreuth, Germany; ^2^Smithsonian Tropical Research Institute, Ancón, Panama; ^3^Department of Agriculture and Natural Resources Sciences, Namibia University of Science and Technology (NUST), Windhoek, Namibia; ^4^Plant Ecology Group, Institute of Evolution and Ecology, University of Tübingen, Tübingen, Germany

**Keywords:** annual species, drylands, growth rate, life-history strategy, leaf structure, rainfall gradient, resource-use strategy, root morphology

## Abstract

The plant economics spectrum hypothesizes a correlation among resource-use related traits along one single axis, which determines species’ growth rates and their ecological filtering along resource gradients. This concept has been mostly investigated and shown in perennial species, but has rarely been tested in annual species. Annuals evade unfavorable seasons as seeds and thus may underlie different constraints, with consequences for interspecific trait-trait, trait-growth, and trait-environment relations. To test the hypotheses of the plant economics spectrum in annual species, we measured twelve resource-use related leaf and root traits in 30 winter annuals from Israel under controlled conditions. Traits and their coordinations were related to species’ growth rates (for 19 species) and their distribution along a steep rainfall gradient. Contrary to the hypotheses of the plant economics spectrum, in the investigated annuals traits were correlated along two independent axes, one of structural traits and one of carbon gain traits. Consequently, species’ growth rates were related to carbon gain traits, but independent from structural traits. Species’ distribution along the rainfall gradient was unexpectedly neither associated with species’ scores along the axes of carbon gain or structural traits nor with growth rate. Nevertheless, root traits were related with species’ distribution, indicating that they are relevant for species’ filtering along rainfall gradients in winter annuals. Overall, our results showed that the functional constraints hypothesized by the plant economics spectrum do not apply to winter annuals, leading to unexpected trait-growth and trait-rainfall relations. Our study thus cautions to generalize trait-based concepts and findings between life-history strategies. To predict responses to global change, trait-based concepts should be explicitly tested for different species groups.

## Introduction

Trait-based schemes, such as the plant economics spectrum, characterize general combinations and trade-offs among functional traits and their relations to environmental conditions (e.g., Grime, 1977; Westoby et al., 2002; Wright et al., 2004; Reich, 2014). They are widely used for assessing and predicting community assembly and ecosystem functioning under current and future conditions (e.g., Westoby et al., 2002; Wright et al., 2004; Reich, 2014). However, the plant economics spectrum has been mainly developed and tested for woody and perennial herbaceous species (reviewed in Reich, 2014), whereas investigations of this concept in annual species are virtually missing (but see Brouillette et al., 2014). The universality of the plant economics spectrum to reflect functional constraints and interspecific trait-environment relations across life-history strategies (annuals vs. perennials) therefore remains unclear.

Annual species substantially contribute to species diversity, primary production, and ecosystem services in many dryland ecosystems worldwide (Noy-Meir, 1973; Tielbörger et al., 2014; Ruppert et al., 2015). These ecosystems have been characterized as particularly vulnerable to global change (Sala et al., 2000; Schröter et al., 2005), underscoring the relevance of assessing and predicting species’ responses to climate and land-use change. Environmental changes may affect annual plant communities even faster than perennial ones due to their short life cycle. Understanding interspecific trait-trait relations and the ecological filtering of annuals is therefore timely and of practical relevance.

The plant economics spectrum hypothesizes an interspecific trade-off between trait attributes conferring rapid resource acquisition (i.e., acquisitive or fast attributes, e.g., high assimilation rate and nutrient concentrations, low tissue density, Table 1) and those conserving resources (i.e., conservative or slow attributes, e.g., low assimilation rate and nutrient concentrations, and high tissue density) at the whole plant level, i.e., among and within leaves, stems, and roots (Reich, 2014). Due to functional constraints to avoid resource shortage or excess, interspecific variation of resource-use related leaf, stem, and root traits should thus be coordinated along one single axis of variation (Freschet et al., 2010; Reich, 2014).

**Table 1:** Studied resource-use related traits, and relative growth rate with their abbreviation (abb.), unit, and hypothesized association with the resource-use strategy according to the plant economics spectrum.

Trait	abb.	Unit	Association with resource-use strategy
Specific leaf area	SLA	mm^2^/mg	a
Leaf dry matter content	LDMC	mg/g	c
Leaf tissue density	LTD	g/cm^3^	c
Leaf thickness	Lthick	mm	c
Area-based photosynthetic rate	A_area_	μmol/(m^2^·s)	a
Area-based nitrogen content	N_area_	mg/mm^2^	a
Mass-based photosynthetic rate	A_mass_	μmol/(g·s)	a
Mass-based nitrogen content	N_mass_	mg/g	a
Mass-based carbon content	C_mass_	mg/g	c
Specific root length	SRL	m/g	a
Root tissue density	RTD	g/cm^3^	c
Root diameter	Rdia	mm	c
Relative growth rate	RGR	g/(g·day)	a

a and c, respectively, indicate whether a high trait value is considered to be associated with an acquisitive or conservative resource-use strategy as the opposite extremes along a continuum.

According to the plant economics spectrum, the trade-off among resource-use related traits should influence species’ growth rates and their ecological filtering along resource gradients (Reich, 2014). Species with acquisitive trait attributes should exhibit high growth rates under high resource availability, but exhibit low performance under resource-poor conditions because of their higher resource demand (Reich, 2014). In contrast, species with conservative trait attributes minimize performance losses under resource-poor conditions (i.e., they exhibit high stress resistance) but at the cost of lower growth rates (Reich, 2014). The resulting interspecific growth-stress resistance trade-off should lead to ecological filtering of species along resource gradients, with acquisitive/fast species predominating under high resource availability, and conservative/slow species under low resource availability (Grime and Hunt, 1975; Reich, 2014).

The interspecific trait-trait, trait-growth, and trait-environment relations expected by the plant economics spectrum have been demonstrated in woody and perennial herbaceous species from various ecosystems (reviewed in Reich, 2014). Only a few studies showed trait-trait or trait-environment relations deviating from the hypotheses of this concept (Baraloto et al., 2010; Fortunel et al., 2012; Kramer-Walter et al., 2016). However, these few studies are significant, because they indicate that the plant economics spectrum may not be universally applicable across different ecosystems, life forms, and/or life-history strategies.

In contrast to perennial species, annuals are characterized by early reproduction at small vegetative size, a short lifespan, high reproductive allocation, and especially annuals from drylands by a pronounced between-year seed dormancy, acting as bet-hedging mechanism against unpredictable reproductive failure (Grime, 1977; Philippi and Seger, 1989; Kooyers, 2015). Additionally, annuals have been assumed and shown to exhibit pronounced acquisitive trait attributes and high growth rates (Grime, 1977; Garnier and Laurent, 1994; Roumet et al., 2006; Kooyers, 2015). This trait combination enables them to evade unfavorable conditions in time, i.e., annuals exhibit an escape strategy (sensu Levitt, 1980; Kooyers, 2015). Although all annuals show this strategy, they differ in their trait attributes and occur in a wide range of environmental conditions (Bilton et al., 2016; Li and Shipley, 2017; Blumenthal et al., 2020). The consequences of annual’s life-history on interspecific trait-trait, and trait-growth relations, and on their ecological filtering along resource gradients, however, remain almost unexplored.

To our knowledge, interspecific studies testing the plant economics spectrum in annuals are missing. Intraspecifically, one study has addressed differences of resource-use related leaf traits along a rainfall and nutrient gradient in an annual desert forb (Brouillette et al., 2014). Populations showed the expected trait-trait correlations, but contrary to the assumptions of the plant economics spectrum, acquisitive trait attributes were associated with low resource availability (dry and nutrient-poor habitats) and conservative attributes with high resource availability (wetter, nutrient-rich habitats; Brouillette et al., 2014). However, to rigorously test the applicability of the plant economics spectrum in annuals, we need multi-species studies that directly link comparative trait assessments across species with their growth rates and their distribution along resource gradients.

In the present study, we addressed this gap and tested interspecific trait-trait, trait-growth, and trait-environment relations in winter annuals from rangelands in Israel. Israel is characterized by a high diversity of annual species (Tielbörger et al., 2014) and by steep rainfall gradients, ranging from arid conditions (short growing season, low, unpredictable rainfall) to mesic-Mediterranean conditions (longer growing season, high, predictable rainfall). The region thus provides an ideal study system to investigate the variation of traits and growth rates across annual species, and to relate them to their distribution along rainfall gradients to assess species’ ecological filtering.

Winter annuals grow and reproduce in the mild, rainy season (winter), and survive the dry, hot season (summer) as seeds, i.e., they escape the dry season. Accordingly in winter annuals, arid (i.e., resource poor) conditions should favor species with pronounced escape traits, which are considered to be associated with acquisitive trait attributes and high growth rates (see above, Grime, 1977; Kooyers, 2015). This trait combination should enable them to grow and reproduce within the short rainfall season. Toward the opposite, moist side of rainfall gradients, resource availability and competition intensity increase, but rainfall season is still interrupted by occasional dry spells (Noy-Meir, 1973; Schiffers and Tielbörger, 2006; Ziv et al., 2014). Under these conditions, annuals with sufficient drought resistance to withstand dry spells in the vegetative and reproductive phase should be favored, since they can utilize the whole length of the growing season to attain larger heights for an increased competitive effect. Higher stress (drought) resistance is associated with conservative trait attributes and slow growth according to the hypotheses of the plant economics spectrum (Reich, 2014). Ecological filtering in winter annuals should favor species with acquisitive (instead of conservative) traits in arid conditions and species with conservative (instead of acquisitive) traits in more mesic Mediterranean conditions. The expected interspecific trait changes along rainfall gradients in winter annuals are thus opposite to the predictions of the plant economics spectrum and the patterns in perennials (Reich, 2014), but consistent with the findings on intraspecific trait variation in an annual forb (Brouillette et al., 2014).

In the present study, we measured twelve traits that are considered relevant for resource-use by the plant economics spectrum (Table 1) in 30 winter annual species from Israel under common, controlled conditions. We analyzed trait-trait relations, as well as the relations of traits to species’ growth rates (for 19 species) and to their distribution across a steep regional rainfall gradient. Specifically, we addressed the following hypotheses:

- Resource-use related leaf and root traits are correlated along one main axis of variation, reflecting a trade-off between acquisitive and conservative trait attributes.
- Species’ growth rates are influenced by their trait combinations, i.e., their positions along the main trait axis. Species with acquisitive traits exhibit high growth rates, while species with conservative traits exhibit low growth rates.
- Species’ distributions along a rainfall gradient are related with their trait combinations and growth rates. Fast-growing annuals with acquisitive traits are associated with arid conditions, whereas slow-growing species with conservative traits are associated with wetter conditions.

## Materials and Methods

### Study System

Israel in the Eastern Mediterranean Basin comprises steep regional rainfall gradients from both East-West (across 50 km) and North-South (across 350 km) with high and more predictable mean annual rainfall (MAR) in mesic-Mediterranean areas in the north and west (up to 800 mm/year ± 18%, mean ± CV) and less, very unpredictable rainfall in the desert in the south (20 mm/year ± 55%) and toward the Dead Sea (east). The length of the rainfall season, which corresponds to the main growing season, as well as primary productivity and competition intensity decrease toward arid conditions, while average temperature hardly changes (Schiffers and Tielbörger, 2006; Tielbörger et al., 2014). The region is characterized by semi-arid shrublands with mostly winter annual species dominating the inter-shrub matrix. They account for up to 90% of species diversity, and between 55% and 99% of net primary production (Tielbörger et al., 2014).

### Study Species and Plant Material

The study focused on 30 winter annual species comprising 22 forbs (including six legumes) and eight grasses (Supplementary Table S1). Species selection considered the following criteria: (1) high abundance in the region, (2) inclusion of several plant families, (3) wide differences in their distribution along the rainfall gradients (based on BioGIS, 2018), and (4) seed availability. The 30 species belonged to 27 genera and seven families, and all had C3 photosynthesis. Seeds were collected in the mid-range of the regional rainfall gradient from natural habitats in two sites that are about 40 km apart from each other (Lahav, N 31°23' E 34°54', 300 mm MAR and Matta, N 31°42' E 35°3', 540 mm MAR; for details see Tielbörger et al., 2014) in April 2012. The sampling comprised at least 50 plants per species distributed in an area of 1.0–1.5 km^2^.

The field-collected seeds were germinated and grown under common conditions with natural light and ample water supply in a greenhouse in Tübingen (Germany) during winter 2013/2014 to produce F1 seeds (inbred lines) with homogenized parental effects. F1 seeds were over-summered for two months (mid-June to mid-August) in a greenhouse in Bayreuth (Germany) to break summer dormancy before the start of the experiments (see Tielbörger et al., 2012). The plants for trait measurements were grown from F1 seeds under common conditions in a greenhouse in Bayreuth (Germany) during winter 2017/2018 (except for growth rate, grown in 2018/2019 under similar conditions). The comparative approach focused on trait differences across species, the level considered in the plant economics spectrum, and allowed to exclude intraspecific trait variation introduced by phenotypic plasticity or ecotypic differentiation.

### Plant Cultivation in the Greenhouse

Plants were germinated and grown in cylindrical pots (1 L volume, diameter 6.5 cm, depth 36 cm, Deepot Cells, Stuewe & Sons, Oregon, United States) with a 1:1 mixture of sand and compost supplemented with 5 g of amorphous silicon (Aerosil 300, Evonik Industries AG, Essen, Germany). Temperature was set to 20–23°C in winter and 20–26°C in spring. Natural light was supplemented by artificial lights, and day length was adapted to natural variation in Israel for unbiased phenology. All plants received water in ample supply and were fertilized several times with Wuxal Super (NPK fertilizer 8–8-6, Wilhelm Haug GmbH & Co) to preclude nutrient limitation. The pots with the different species were randomly distributed in the greenhouse and rearranged every two weeks. Plants were grown until the end of their life cycle (31–34 weeks after sowing) indicated by leaf senescence in most of the species.

### Trait Measurements

Twelve resource-use related traits, including leaf and root traits as well as structural and carbon gain related traits, were measured on 5–14 individuals per species (Supplementary Table S2). Leaf traits were assessed on one randomly chosen, healthy, mature leaf per plant 8–12 weeks after sowing. Root traits were measured 14–20 weeks after sowing.

To determine specific leaf area (SLA = LA/DW) and leaf dry matter content (LDMC = DW/FW, LDMC is the inverse of leaf water content, LWC in mg/g, LWC = 1,000-LDMC), we measured saturated fresh weight (FW) of the leaf after hydrating plants overnight (approx. 15 h), and dry weight (DW) after oven-drying. Leaf area (LA) was quantified with an Area-meter (Model LI 3100, Li-Cor Bioscience, Lincoln, NE, United States). Leaf thickness (Lthick) was measured with a micrometer (Mitutoyo M110-25, graduation 0.01 mm) at three points in the center of the leaf blade, avoiding the midrib and primary veins, and averaged. Leaf tissue density (LTD) was calculated as ratio of dry weight to leaf volume (leaf volume = LA·Lthick).

Maximum photosynthetic rate per leaf area (A_area_) was measured with an infra-red gas analyzer Li-Cor 6400 (Li-Cor, Lincoln, NE, United States) between 8.30 and 11.00 h at a light intensity of 2000 μmol·photons/(m^2^·s^1^) (based on light response curves for a species subset), 25°C, and 400 ppm CO_2_. If the leaf did not fill the measurement chamber, photosynthetic rate was re-calculated based on leaf area measurements with an Area Meter (see above). Usually, one leaf per individual was measured, but in species with very small or thin leaves (e.g., a few grasses, *Filago*, and *Helianthemum*) several leaves were jointly arranged in the measurement chamber. The leaves used for photosynthesis measurements (few exceptions in *Psilurus incurvus*, *Rostraria cristata*) were oven-dried and ground to analyze their mass-based nitrogen content (N_mass_) and carbon content (C_mass_) with an EA-IRMS coupling (Elemental Analyzer NA 1108, CE Instruments, Milan, Italy; Interface ConFlo III, Finnigan MAT, Bremen, Germany; Isotope ratio mass spectrometer: delta S, Finnigan MAT, Bremen, Germany). Photosynthetic rate and nitrogen content were converted to their area- or mass-based equivalent (A_mass_, N_area_) *via* SLA based on species’ average values.

Morphological root traits were determined on three subsamples of fine roots (diameter < 2 mm, stored in 35% ethanol before the measurements) from the upper, middle, and lower part of the root (except whole roots in *Filago* and *Helianthemum salicifolium*). The root samples were stained with toluidine blue (0.2 g/l) and scanned (Scanner Epson Perfection V800/V850 photo scanner, 600 dpi). Images were analyzed with WinRHIZO © Reg 2017 (Regent Instruments Inc., Quebec, Canada) to determine mean diameter (Rdia), volume, and length of the scanned root sample. Samples were oven-dried to measure dry weight, and to calculate specific root length (SRL = root length/root dry weight) and root tissue density (RTD = root mass/root volume). Calculations of SRL and RTD considered diameter heterogeneity by using summed root length and volume from 40 diameter classes (see Rose, 2017), respectively.

Species’ average relative growth rate (RGR) was assessed in 19 species (see Supplementary Table S1) in a separate plant set that was grown under similar conditions (see Plant cultivation) in winter 2018/19. RGR was calculated based on species-specific averages of aboveground biomass (AB) in week 16 (t1) and week 22–23 (t2) after sowing as: RGR = (AB2–AB1)/[AB1 · (t2-t1)].

### Species’ Distribution Along Rainfall Gradients

Species’ distribution along the regional rainfall gradients in Israel was characterized based on their occurrences (presence/absence data) in independent biological records (BioGIS, 2018). The BioGIS database provides the mean annual rainfall niche of each species, which is modeled as the average of local mean annual rainfall across all occurrence sites of a respective species. Our study species covered mean annual rainfall niches between 120 mm/year (association with arid conditions) and 580 mm/year (association with Mediterranean conditions, Supplementary Table S1).

### Statistical Analyses

Trait differences across species were tested with *F*-tests on linear models (LM) with species’ identity as explanatory factor. Traits (except Rdia and C_mass_) were natural log-transformed to improve normality and homoscedasticity. Pairwise trait correlations were calculated with Spearman rank correlation coefficients based on species’ average values.

The main axes of correlations among the resource-use related traits were assessed with a principal component analysis (PCA) at the plant level (i.e., combining leaf and root traits) based on species’ average trait values. Since mass- but not area-based traits are explicitly included in the leaf economics spectrum (Wright et al., 2004), we equivalently considered only A_mass_, N_mass_, and C_mass_ in the PCA. The main trait correlations though were similar between a PCA with these mass-based traits and a PCA considering instead A_area_, N_area_, and C_area_ (Pearson correlation coefficients among species’ scores along the trait axes |0.48| ≤ r ≤ |0.87|). We also calculated PCAs separately for leaf and for root traits. The trait coordinations in leaf and root traits were similar to those observed at the whole plant level (Supplementary Table S3). Species’ scores along the principal components (PC, in the following referred to as trait axes) calculated for the whole plant level were therefore used to characterize species’ trait combinations.

We tested for differences among life forms (i.e., grasses, non-legume forbs, legumes) in species’ scores along the main trait axes (PC 1, PC 2) with *F*-tests on LMs separately calculated for each trait axis and Tukey *post-hoc* tests. Additionally, the main axes of trait correlations were separately assessed for forbs (including legumes) and grasses with PCAs as described above for all species. Trait correlations within life forms were similar to the ones among all species (Pearson correlation coefficients among species’ scores along the trait axes |0.60| ≤ r ≤ |0.98|). The relations between species’ scores along the trait axes or single traits, species’ growth rate and distribution were therefore calculated for the whole species set.

**Figure 1:**
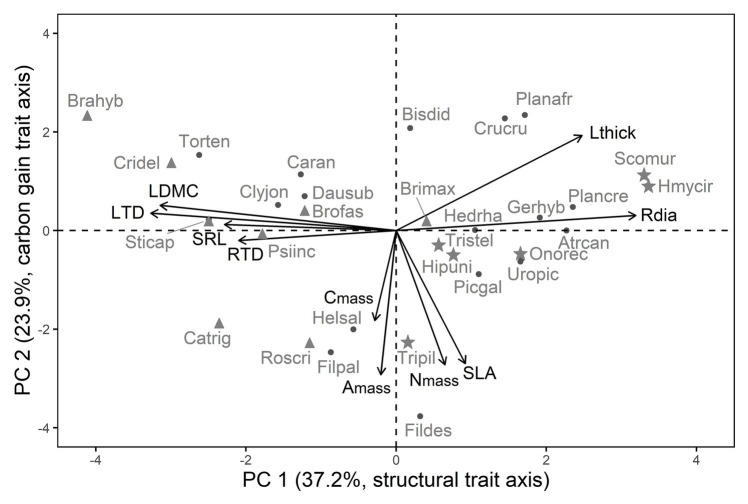
Resource-use related traits are correlated along two axes in 30 winter annual species, the first axis corresponds to structural traits (PC 1), and the second to carbon gain traits (PC 2). Trait abbreviations and trait loadings are given in Tables 1, 2, and species abbreviations are given in Supplementary Table S1, respectively. Symbols indicate life form: triangles for grasses, points for non-legume forbs, asterisks for legumes. Grasses differed from non-legume forbs and legumes in their species’ scores along PC 1, but not along PC 2.

The relations between species’ scores along the main trait axes (PC 1, PC 2, i.e., species’ trait combinations) and their relative growth rates were tested with *F*-tests on LMs separately calculated for each trait axis. The relations between the species’ scores along the trait axes and relative growth rates did not qualitatively differ depending on whether the species’ scores were derived from a PCA with the full species set (30 species, Figure 1) or from a PCA with the species set with growth rate measurements (19 species, Supplementary Figure S1). For consistency among all analyses, we therefore presented the findings with the PCA based on the full species set. Relations between single traits and relative growth rates were also tested with *F*-tests on LMs separately calculated for each trait. Relative growth rates were natural log-transformed in the LM with C_mass_ to improve normality and homoscedasticity.

The relations of species’ scores along the main trait axes (PC 1, PC 2, i.e., species’ trait combination) or single traits with species’ distribution along the rainfall gradient (i.e., mean annual rainfall niche) were tested with *F*-tests on LMs separately calculated for each axis or trait. Relations were also calculated with minimum and maximum rainfall niche based on BioGIS (2018), but qualitative results hardly changed (results not shown).

Adjusted significance level according to Holm-Bonferroni sequential correction (Holm, 1979; Gaetano, 2013) was applied to the multiple tests for species’ differences in the traits, and for trait (axes) relations to species’ growth rates and distribution. Since we tested only pre-planned hypotheses (see Table 1), we interpreted the results based on unadjusted significance level (see Armstrong, 2014).

All analyses were conducted with R 3.6.1 (R Core Team, 2019).

## Results

The twelve resource-use related traits differed significantly across the 30 winter annuals, with almost 2–5 fold variation, and in relative growth rate with about 26 fold variation, respectively (*F*-values between 2.4 and 93.2, all *p* < 0.001, Supplementary Table S2). The attribute range of several leaf traits in the studied annuals almost corresponded to 60–80% of the globally documented trait ranges in plant species from different ecosystems and life forms worldwide (Kattge et al., 2020, see details in Supplementary Table S2). In all traits, the values of the investigated annuals fall within the acquisitive end of the global trait range (e.g., high SLA, N_mass_, Supplementary Table S2).

Traits were correlated along two PCs, instead of one as we had expected (Figure 1; Table 2). PC 1 corresponded in positive direction with Rdia and Lthick, and in negative direction with LDMC, SRL, RTD, and LTD (Figure 1; Table 2). PC 1 thus summarized structural leaf and root traits (in the following referred to as structural trait axis). PC 2 was highly negatively correlated with A_mass_, N_mass_, C_mass_, and SLA, four traits associated with carbon gain (in the following referred to as carbon gain trait axis, Figure 1; Table 2). The independence between structural and carbon gain traits was also reflected in the pairwise trait correlations (Supplementary Table S4).

**Table 2:** Trait loadings on the first two principal components (PC) of a principal component analysis (PCA) with ten resource-use related traits in 30 winter annual species (see Figure 1).

	PC 1	PC 2
Eigenvalue	3.71	2.39
Explained variance [%]	37.1	23.9
LTD	**−0.47**	0.06
LDMC	**−0.45**	0.09
Rdia	**0.46**	0.06
Lthick	0.36	0.35
SRL	−0.33	0.02
RTD	−0.30	−0.04
SLA	0.13	**−0.49**
N_mass_	0.09	**−0.49**
C_mass_	−0.04	−0.33
A_mass_	−0.03	**−0.53**

The table shows the eigenvalues, the proportion of explained variance of both PCs, and the loadings of the traits. Traits were ordered according to their |loading| on PC 1. Trait loadings > |0.4| are marked in bold.

Grasses differed from non-legume forbs and legumes in their scores along the structural trait axis (PC 1, LM statistics F_2,27_ = 11.23, *p* < 0.001, *R*^2^ = 0.41) with higher LTD and LDMC in grasses, but not along the carbon gain axis (PC 2, F_2,27_ = 0.12, *p* = 0.89, Figure 1). Non-legume forbs and legumes exhibited similar scores along both trait axes (Figure 1). The trait correlations along PC 1 and PC 2 within each life form (i.e., forbs vs. grasses) were similar to the ones among all species (Supplementary Figure S2).

**Figure 2:**
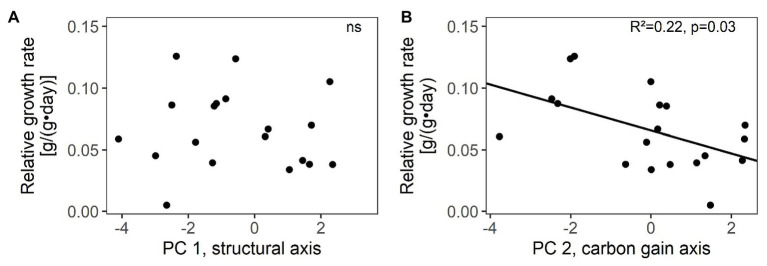
Relations between relative growth rates and species’ scores along the two main trait axes of resource-use related traits: **(A)** structural trait axis (PC 1) and **(B)** carbon gain trait axis (PC 2) in 19 winter annual species. *R*^2^ values and significance are given (ns not significant, for details see Supplementary Table S5). Species’ scores were based on the PCA in Figure 1 with 30 species.

Species’ scores along the carbon gain trait axis (PC 2) were related to relative growth rate, i.e., species with higher A_mass_, N_mass_, C_mass_, and SLA grew faster, but scores on the structural trait axis were unrelated to relative growth rate (Figure 2; Supplementary Table S5). Among single traits, only A_mass_ and A_area_ were positively related with relative growth rate (Supplementary Table S5).

**Figure 3:**
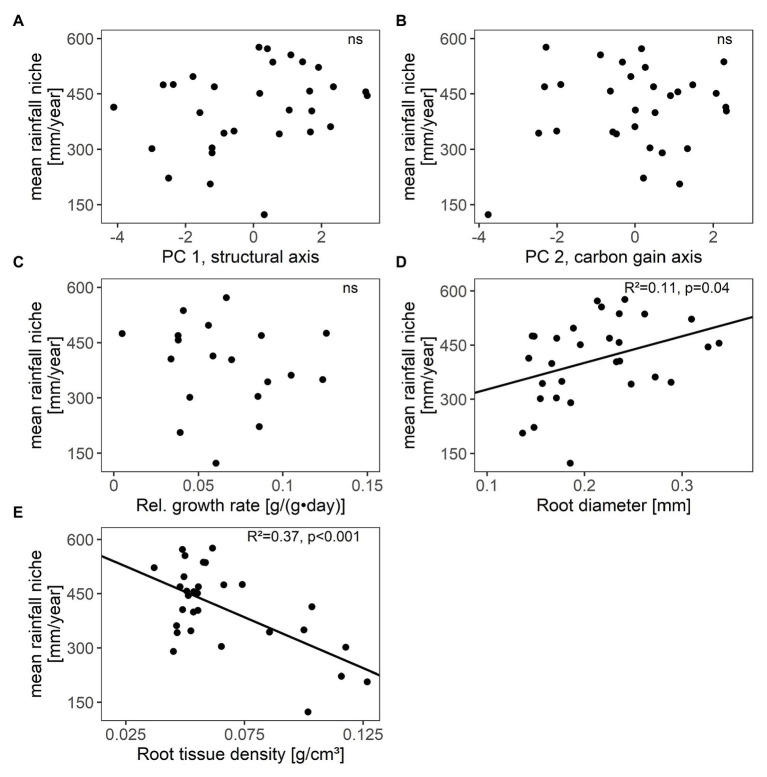
Relations between species’ mean annual rainfall niche (i.e., species’ distribution along the rainfall gradient) and **(A,B)** their scores along the two main axes of resource-use related traits (PC 1, PC 2), **(C)** relative growth rate, **(D)** root diameter, and **(E)** root tissue density in 30 winter annual species (relative growth rate was only assessed in 19 species). *R*^2^ values and significance were given (ns not significant, for details see Supplementary Table S6). Further single traits were unrelated to species’ mean annual rainfall niche (see Supplementary Table S6).

Species’ scores along the carbon gain and structural trait axes (PC 1, PC 2) as well as relative growth rates were independent from their distribution along the rainfall gradient (mean annual rainfall niche; Figures 3A–C; Supplementary Table S6). However, among single traits, root traits were related with species’ distribution. Species with higher RTD and smaller Rdia were associated with arid conditions (Figures 3D,E; Supplementary Table S6).

## Discussion

The main premise of the plant economics spectrum is that resource-use related traits are correlated along a single axis, comprising both structural and carbon gain traits, as well as traits of different plant organs (Reich, 2014). In contrast, in the investigated winter annuals structural and carbon gain traits were decoupled, and correlated along two independent axes. The unexpected decoupling contradicts the predictions of the plant economics spectrum and had pervasive consequences on trait relations to growth rates and species’ distributions along the rainfall gradient that were also inconsistent with the hypotheses of the plant economics spectrum.

### Trait-Trait Relations: Structural and Carbon Gain Traits Are Independent

The decoupling between structural and carbon gain traits we found in the investigated winter annuals has, to our knowledge, not been observed in perennial herbaceous or woody species. Instead, perennial herbaceous or woody species predominantly show the expected correlation of resource-use related traits along a single axis (e.g., Freschet et al., 2010; Liu et al., 2010; de la Riva et al., 2016a). In perennials, variation of leaf structure, especially of leaf tissue density and thickness, is mainly due to differences in carbon content (Roderick et al., 1999; de la Riva et al., 2016b). Higher carbon contents are associated with a higher proportion of sclerenchyma, thicker mesophyll layers and/or thicker mesophyll cell walls (de la Riva et al., 2016b; Onoda et al., 2017). This leaf structure leads to lower mass-based photosynthetic rate due to higher diffusion resistance to carbon dioxide, shading of chloroplasts, or lower proportion of mass-based nitrogen content (Niinemets, 1999; Shipley et al., 2005; Onoda et al., 2017). In the studied winter annuals, however, structural trait variation (i.e., the structural trait axis) was independent from carbon content. The unexpected decoupling between structural and carbon gain traits implies that structural trait variation is due to components that do not constrain photosynthetic rate.

Our study species exhibited high water contents, leaf hairiness, and silicon accumulation. These traits are assumed to increase species’ ability to cope with the environmental conditions in drylands by maximizing water storage, reflecting sunlight, decreasing transpiration, and deterring grazing herbivores (Ehleringer and Mooney, 1978; Woodman and Fernandes, 1991; Sack et al., 2003; Katz, 2019). They have additionally been proposed to dilute the relations between mass-based nitrogen content and structural leaf traits expected by the leaf economics spectrum (Grubb, 2016). Consistently, they influenced leaf tissue density and thickness in our study species. Leaf tissue density was strongly influenced by silicon instead of carbon content (Supplementary Table S4; Supplementary Method 1); and in forbs, high tissue density was additionally associated with long and/or dense leaf hairs (pers. obs.). Similarly, high leaf thickness mainly resulted from high water content and was negatively associated with carbon content (Supplementary Table S4). Despite their influence on leaf structure, silicon content, leaf water content, and leaf hairiness did not affect mass-based photosynthetic rate, since they did not constrain the proportion of mass-based nitrogen content (Supplementary Table S4), or the diffusion resistance to carbon dioxide. Decoupling between structural and carbon gain traits, which is in contrast to the hypothesis of the plant economics spectrum, thus might not be restricted to winter annuals, but should also emerge in other species groups, in which traits other than carbon content lead to leaf structure variation, e.g., in perennial grasses with high silicon accumulation or succulent perennials (compare to Grubb, 2016).

Despite the decoupling of structural and carbon gain traits, the structural traits of roots and leaves were correlated in the studied winter annuals (SRL, RTD, Rdia, and LTD, LDMC, Lthick, respectively), consistent with findings in perennials (Freschet et al., 2010; Reich, 2014; de la Riva et al., 2016a). The structural analogy between leaves and roots thus is not a consequence of the correlation of resource-use related traits along one axis, but might result from tissues pervading the entire plant, such as xylem and phloem vessels (Wahl and Ryser, 2000; Hummel et al., 2007).

### Trait-Growth Relations: Growth Rate Is Independent From Structural Traits

The plant economics spectrum proposes that species’ positions along the single axis of resource-use related traits determine their growth rates (Reich, 2014). Consistent with this hypothesis, winter annuals with higher carbon gain trait attributes exhibited higher relative growth rates. However, structural traits were unrelated to relative growth rate, reflecting their uncoupling from carbon gain. Winter annuals with a wide variation of structural traits thus exhibited similar relative growth rates in contrast to the findings in perennials and the hypothesis of the plant economics spectrum (Lambers and Poorter, 1992; Reich, 2014).

The structural traits should, however, influence species’ stress resistance. In our study species, the structural trait axis was associated with silicon content and leaf hairiness (see above), traits assumed to deter grazing herbivores (Woodman and Fernandes, 1991; Katz, 2019), as well as with turgor loss point, i.e., the water potential at which leaves lose turgor (Supplementary Figure S3; Supplementary Method 1). Turgor loss point is considered as a major physiological determinant of species’ drought response (Bartlett et al., 2012; Sun et al., 2020). A relation between structural traits and species’ drought and grazing resistance has also been indicated across life forms in semi-arid ecosystems (Blumenthal et al., 2020).

Independence between growth rate and structural traits thus implies that the assumptions of the growth-stress resistance trade-off (see Grime and Hunt, 1975; Reich, 2014) do not apply to the studied winter annuals. Growth rates indeed turned out to be independent of species’ stress resistance (to grazing and drought) in our study species (Kurze et al., in review). Similar findings emerged in a few studies of perennial herbaceous species (Fernández and Reynolds, 2000; Jung et al., 2020).

### Trait-Environment Relations: Species’ Filtering Along the Rainfall Gradient Is Only Reflected in Root Traits

We expected that arid conditions favor winter annuals with acquisitive trait attributes and high growth rates due to their ability to reproduce within a short period and thus to escape drought (see Levitt, 1980; Kooyers, 2015). However, in the investigated annuals, species’ distribution along the rainfall gradient was unrelated to their scores along the structural and carbon gain trait axis and to growth rate. It is improbable that this unanticipated result is due to our focus on trait variation across species, which did not consider ecotypic variation. Ecotypic trait variation is usually considerably smaller than interspecific variation (Garnier et al., 2001; Kazakou et al., 2014; Siefert et al., 2015). This has been also shown in some of our study species (Bergholz et al., 2017; Kurze et al., 2017; Álvarez-Cansino et al., unpublished data). Additionally, at the intraspecific level, resource-use related leaf traits did not show directional changes along the rainfall gradient in our study system (Bergholz et al., 2017; Kurze et al., 2017).

Rather, this unexpected finding likely emerged from the independence of growth rate and stress resistance, which facilitates similar ecological success of species with alternative trait combinations and supports the co-occurrence of annuals with a wide range of structural and carbon gain traits and growth rates along the rainfall gradient. Theoretical models (Marks and Lechowicz, 2006) and studies on interspecific variation of leaf traits in woody and perennial herbaceous species support that different trait combinations can be successful in the same environment (Wright et al., 2004; Cernusak et al., 2011; Forrestel et al., 2017; Muir et al., 2017). In our study species, the structural trait axis was associated with traits related to both drought and grazing resistance (e.g., turgor loss point, leaf silicon content, leaf hairiness, see above), which should be differentially filtered along the rainfall gradient and may offset each other (Carmona et al., 2012; Rota et al., 2017). Similarly, fast and slow growth rates may confer species a high competitive effect and high competitive response under wetter conditions (Goldberg and Landa, 1991; Liancourt et al., 2009). Consequently, contrary to the expectations of the plant economics spectrum, neither species’ trait combinations along the structural or carbon gain trait axis nor growth rates led to ecological filtering along the rainfall gradient in winter annuals.

Nevertheless, fine root traits were associated with species’ distribution along the rainfall gradient in the studied annuals. Fine root traits should be more directly related to species’ drought resistance than the combination of structural traits (reflected in the structural trait axis), which comprised traits of both drought and grazing resistance. Root traits have indeed previously been shown to be more strongly related to the drought survival of annual species than leaf traits (Harrison and LaForgia, 2019). In our study species, root diameter decreased and root tissue density increased with increasing species’ association to arid conditions. Species with these trait attributes are assumed to exhibit higher drought resistance, since thin and dense roots decrease the resistance to radial water inflow and are less prone to cavitation due to smaller xylem vessels (Wahl and Ryser, 2000; Comas et al., 2013). Woody species with these root trait attributes have been shown to be favored under low water availability (Nicotra et al., 2002; de la Riva et al., 2018). The root trait changes along the rainfall gradient observed in our study species thus imply that the low water availability during the growing season imposes an ecological filter under arid conditions. Arid conditions thus favor winter annuals with high drought resistance in the vegetative phase conferred by root traits but not with acquisitive traits or high growth rate to escape drought, as we initially expected.

## Conclusion

Our findings showed that the functional constraints hypothesized by the plant economics spectrum do not apply to winter annuals. Winter annuals can thus not be ranked along a single axis of resource-use related traits from acquisitive (or fast) to conservative (or slow), as proposed by the plant economics spectrum (Reich, 2014). The limited applicability of the plant economics spectrum to winter annuals cautions to generalize functional constraints, trait-growth, or trait-environment relations among life-history groups. Explicitly testing and establishing these relations for species groups that may be subjected to different constraints is a precondition for using trait-based approaches to understand and predict species’ performance, community composition, or ecosystem functioning.

## Data Availability Statement

The raw data supporting the conclusions of this article will be made available by the authors, without undue reservation.

## Author Contributions

SK conceived the ideas for this manuscript, and LÁ-C, BE, SK, MB, and KT designed the study. SK coordinated and conducted the trait measurements, analyzed the data, and wrote the manuscript with contributions by LÁ-C and BE. All co-authors commented on subsequent drafts of the manuscripts and gave final approval for publication.

### Conflict of Interest

The authors declare that the research was conducted in the absence of any commercial or financial relationships that could be construed as a potential conflict of interest.
