# Supplementary material for: Rethinking the Plant Economics Spectrum for Annuals: A Multi-Species Study
Source: Front Plant Sci. 2021 Mar 26;12:640862. doi: 10.3389/fpls.2021.640862 (PMC8034396; doi:10.3389/fpls.2021.640862)
Supplement: Supplementary file 1 [file Data_Sheet_1.docx]

Supplementary Material

**Supplementary Figure S1.** Trait correlations in the subset of 19 winter annual species for which relative growth rates were measured based on a principal component analysis.

**Supplementary Figure S2.** Trait correlations separately analysed in forbs and grasses based on principal component analyses.

**Supplementary Figure S3.** Relation between species’ positions along the structural trait axis (PC 1) and turgor loss point.

**Supplementary Table S1.** List of the 30 studied winter annual species from Israel with their abbreviation, family, and mean annual rainfall niche.

**Supplementary Table S2.** Attributes of the resource-use related traits, relative growth rate, and leaf silicon content across the 30 studied winter annual species, and comparison with the global trait range.

**Supplementary Table S3.** Trait correlations separately analysed for leaf and for root traits in 30 winter annual species based on principal component analyses.

**Supplementary Table S4.** Pairwise correlations among the twelve resource-use related traits and leaf silicon content based on Spearman rank correlation coefficients in 30 winter annual species.

**Supplementary Table S5.** Relations between species’ relative growth rate and species’ scores along the main trait axes (PC 1, PC 2), or single traits in 19 winter annual species.

**Supplementary Table S6.** Relations between species’ mean annual rainfall niche and species’ scores along the main trait axes (PC 1, PC 2), relative growth rate, or single traits.

**Supplementary Method 1**. Measurement of leaf silicon content and turgor loss point.

# Supplementary Figures


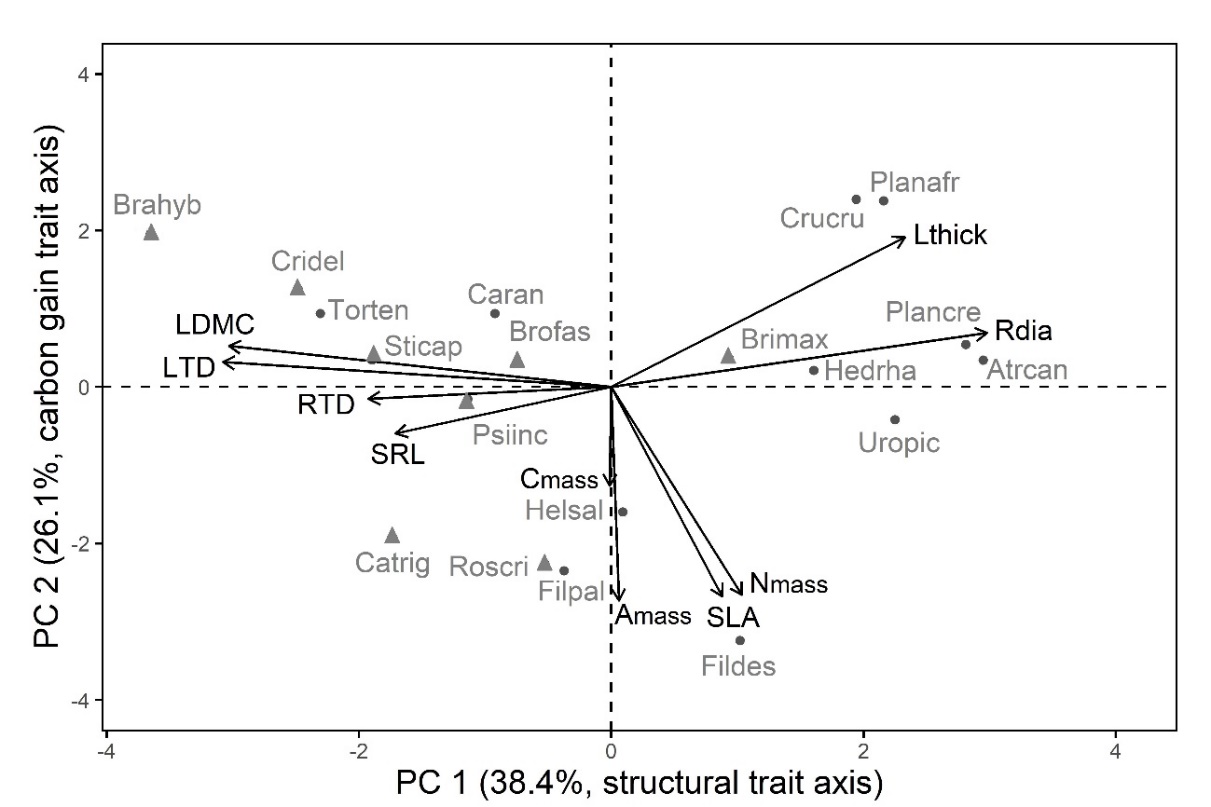


**Supplementary Figure S1.** Trait correlations in the subset of 19 winter annual species for which relative growth rates were measured based on a principal component analysis. Symbols indicate life form: points for forbs, triangles for grasses. Species’ scores along the main trait axes were correlated with species’ scores along the main trait axes in the PCA with the full species set (n=30, Figure 1, Table 2, Pearson correlation coefficient r≥|0.99|), indicating that trait coordinations were similar between both species sets. See Table 1 and Supplementary Table S1 for trait and species abbreviations, respectively.


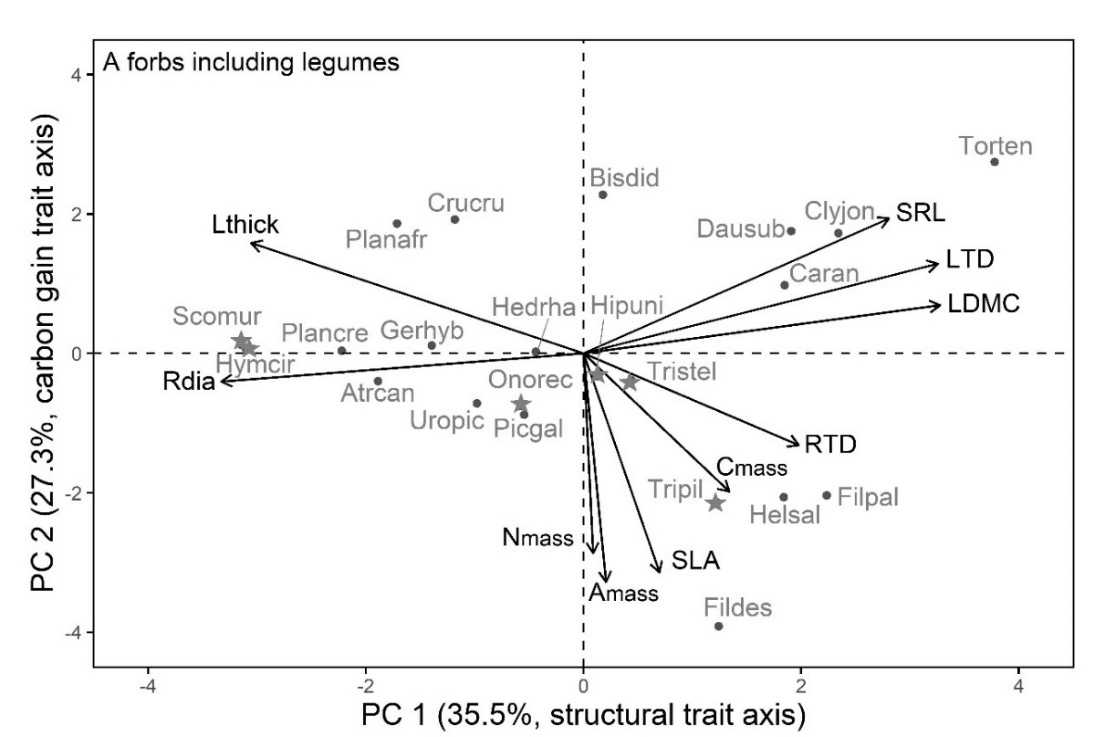

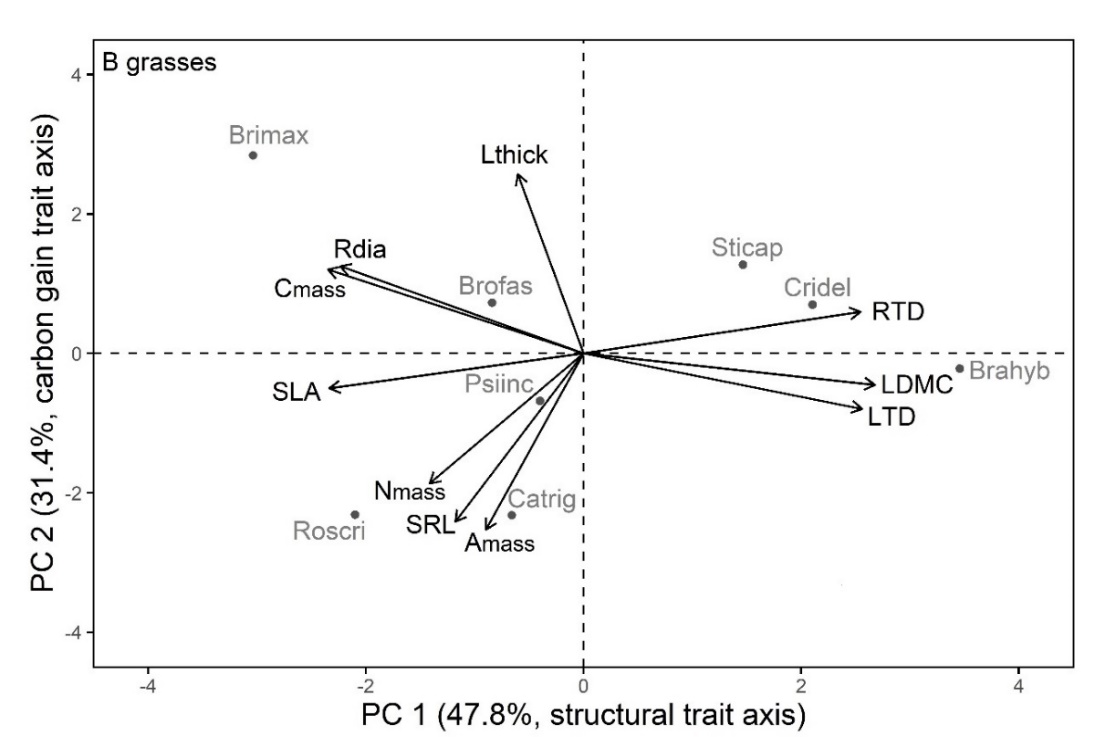


**Supplementary Figure S2.** Trait correlations separately analyzed in (A) forbs (22 species, including 6 legumes, indicated by asterisks) and (B) grasses (8 species) based on principal component analyses. In both life forms, traits were correlated along a structural trait axis (PC 1) and carbon gain trait axis (PC 2) as in the whole species set. See Table 1 and Supplementary Table S1 for trait and species abbreviations, respectively.


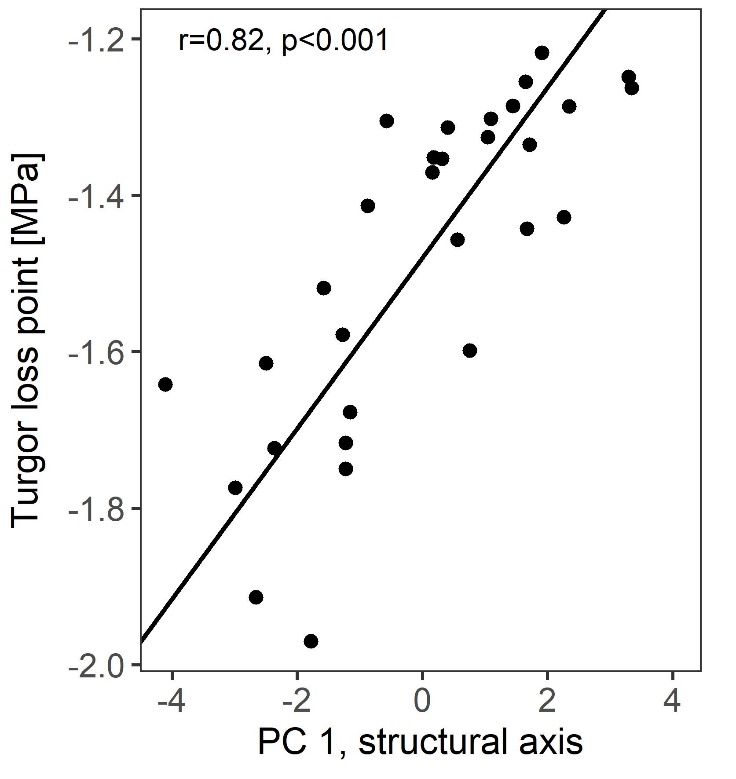


**Supplementary Figure S3.** Relation between species’ scores along the structural trait axis (PC 1) and turgor loss point (see Supplementary Method 1). Relation was analysed with Spearman rank correlation coefficient (r).

# Supplementary Tables

**Supplementary Table S1.** List of the 30 studied winter annual species from Israel with their abbreviation, family, and mean annual rainfall niche (from BioGIS, 2018). The 19 species with relative growth rate measurements are marked with *. Species are ordered alphabetically.

| Species name | abbreviation | family | rainfall niche [mm/year] |
| --- | --- | --- | --- |
| *Atractylis cancellata** | Atrcan | Asteraceae | 361.2 |
| *Biscutella didyma* | Bisdid | Brassicaceae | 450.9 |
| *Brachypodium hybridum** | Brahyb | Poaceae | 413.5 |
| *Briza maxima** | Brimax | Poaceae | 572 |
| *Bromus fasciculatus** | Brofas | Poaceae | 303.7 |
| *Carrichtera annua** | Caran | Brassicaceae | 206 |
| *Catapodium rigidum** | Catrig | Poaceae | 475 |
| *Clypeola jonthlaspi* | Clyjon | Brassicaceae | 398.8 |
| *Crithopsis delileana** | Cridel | Poaceae | 301.5 |
| *Crupina crupinastrum** | Crucru | Asteraceae | 536.7 |
| *Daucus subsessilis* | Dausub | Apiaceae | 290.1 |
| *Filago desertorum** | Fildes | Asteraceae | 122.8 |
| *Filago palaestina** | Filpal | Asteraceae | 343.5 |
| *Geropogon hybridus* | Gerhyb | Asteraceae | 521.7 |
| *Hedypnois rhagadioloides** | Hedrha | Asteraceae | 405.6 |
| *Helianthemum salicifolium** | Helsal | Cistaceae | 349.4 |
| *Hippocrepis unisiliquosa* | Hipuni | Fabaceae | 341.8 |
| *Hymenocarpos circinnatus* | Hymcir | Fabaceae | 444.7 |
| *Ononis reclinata* | Onorec | Fabaceae | 346.9 |
| *Picris galilaea* | Picgal | Asteraceae | 555.3 |
| *Plantago afra** | Planafr | Plantaginaceae | 403.4 |
| *Plantago cretica** | Plancre | Plantaginaceae | 468.9 |
| *Psilurus incurvus** | Psiinc | Poaceae | 496.8 |
| *Rostraria cristata** | Roscri | Poaceae | 468.9 |
| *Scorpiurus muricatus* | Scomur | Fabaceae | 455.2 |
| *Stipa capensis** | Sticap | Poaceae | 221.7 |
| *Torilis tenella** | Torten | Apiaceae | 474.2 |
| *Trifolium pilulare* | Tripil | Fabaceae | 576.1 |
| *Trifolium stellatum* | Tristel | Fabaceae | 535.9 |
| *Urospermum picroides** | Uropic | Asteraceae | 457 |

**Supplementary Table S2**. Attributes of the resource-use related traits, relative growth rate, and leaf silicon content (Si_mass_, see Supplementary Method 1) across the 30 studied winter annual species, and comparison with the global trait range. Given are the mean ± standard deviation (std) across species, minimum (min) and maximum (max) species’ mean, their x-fold variation (var = max/min), and the number of replicates measured in each species (n). Given are also the results of linear models testing for species’ differences in each trait (den. d.f., F-value, p-value and R^2^). Traits significantly differed across species (all p<0.001), and species’ differences in all traits remained significant after Holm-Bonferroni correction. Global trait ranges based on the TRY database (Kattge et al., 2020) are given with minimum (min) and maximum (max) species’ means (min and max represent the 2.5 and 97.5 percentile of global trait variation, respectively), their x-fold variation among species (var) as well as the percentage of the global trait range covered by the investigated annuals (cov. annuals [%] = (trait range annuals/trait range global)*100). See Table 1 for trait abbreviations.

| Trait | unit | 30 annual species | | | | | | | | | global data set | | | cov. annuals |
| --- | --- | --- | --- | --- | --- | --- | --- | --- | --- | --- | --- | --- | --- | --- |
|  |  | mean±std | min | max | var | n | den. d.f. | F | p | R² | min | max | var |  |
| SLA | mm^2^/mg | 33.51±9.34 | 20.65 | 57.56 | 2.8 | 9-14^a^ | 323 | 12.27 | <0.001 | 0.48 | 4.04 | 45.9 | 11.4 | 88.4 |
| LDMC | mg/g | 129.36±46.27 | 66.87 | 291.32 | 4.4 | 9-14^a^ | 323 | 27.14 | <0.001 | 0.68 | 121 | 518 | 4.3 | 58.0 |
| LTD | g/cm^3^ | 0.17±0.07 | 0.08 | 0.30 | 4.0 | 9-14^a^ | 323 | 17.73 | <0.001 | 0.58 | 0.13 | 0.64 | 5.0 | 44.1 |
| Lthick | mm | 0.23±0.09 | 0.12 | 0.47 | 3.8 | 9-14^a^ | 323 | 43.42 | <0.001 | 0.78 | 0.11 | 0.73 | 6.9 | 54.8 |
| A_area_ | μmol/(m^2^•s) | 14.41±4.42 | 6.51 | 25.64 | 3.9 | 7-14^a^ | 292 | 7.32 | <0.001 | 0.36 | 2.07 | 28.89 | 14.0 | 71.2 |
| N_area_ | mg/mm^2^ | 0.001±0.0003 | 0.0006 | 0.0018 | 2.7 | 1 | - | - | - | - | 0.0006 | 0.004 | 6.7 | 35.4 |
| A_mass_ | μmol/(g•s) | 0.46±0.13 | 0.25 | 0.76 | 3.1 | 1 | - | - | - | - | 0.02 | 0.45 | 23.5 | 119.4 |
| N_mass_ | mg/g | 34.23±6.68 | 23.17 | 50.47 | 2.2 | 5-9^a^ | 173 | 2.41 | <0.001 | 0.17 | 8.10 | 41.27 | 5.1 | 81.4 |
| C_mass_ | mg/g | 397.13±22.66 | 355.33 | 444.50 | 1.3 | 5-9^a^ | 173 | 3.34 | <0.001 | 0.25 | - | - | - | - |
| SRL | m/g | 579.46±231.86 | 211.54 | 1105.18 | 5.2 | 8-9^a^ | 208 | 32.34 | <0.001 | 0.79 | 245 | 27722 | 113.2 | 3.3 |
| RTD | g/cm³ | 0.07±0.02 | 0.04 | 0.13 | 3.4 | 8-9^a^ | 208 | 29.04 | <0.001 | 0.77 | 0.067 | 0.697 | 10.4 | 14.3 |
| Rdia | mm | 0.21±0.06 | 0.14 | 0.34 | 2.5 | 8-9^a^ | 208 | 93.16 | <0.001 | 0.92 | 0.17 | 1.32 | 7.7 | 17.5 |
| RGR^b^ | g/(g•day) | 0.07±0.03 | 0.005 | 0.126 | 25.8 | 1 | - | - | - | - | - | - | - | - |
| Si_mass_ | mg/g | 16.98±20.01 | 1.89 | 91.69 | 48.5 | 7-11^a^ | 213 | 191.53 | <0.001 | 0.96 | - | - | - | - |

^a^ exceptions are *Daucus subsessilis* (n=4-6), *Ononis reclinata* (n=5-8), ^b^ for 19 species

**Supplementary Table S3.** Trait correlations separately analyzed for leaf and for root traits in 30 winter annual species based on principal component analyses. Given are the trait loadings on the first two principal components (PC) of PCAs either calculated with the seven resource-use related leaf traits or the three resource-use related root traits. The table shows the eigenvalues, the proportion of explained variance of both PCs, and the loadings of the traits. Traits were ordered according to their |loading| on PC 1. Species’ scores along both PCs of leaf traits and along PC 1 of root traits were highly correlated (Pearson correlation coefficient r>|0.81|) with the species’ scores along the PCs of the PCA calculated for the whole plant level (Figure 1, Table 2).

|  | PC 1 | PC 2 |
| --- | --- | --- |
| Eigenvalue | 2.50 | 2.34 |
| Explained variance [%] | 35.7 | 33.4 |
| Leaf traits | | |
| LDMC | -0.55 | -0.23 |
| LTD | -0.54 | -0.26 |
| SLA | 0.41 | -0.31 |
| N_mass_ | 0.35 | -0.36 |
| A_mass_ | 0.24 | -0.47 |
| Lthick | 0.22 | 0.55 |
| C_mass_ | 0.06 | -0.35 |
| Root traits | | |
| Eigenvalue | 1.92 | 1.03 |
| Explained variance [%] | 64.0 | 34.4 |
| Rdia | -0.71 | 0.0 |
| SRL | 0.52 | 0.67 |
| RTD | 0.47 | -0.74 |

**Supplementary Table S4.** Pairwise correlations among the twelve resource-use related traits and leaf silicon content (Si_mass,_ see Supplementary Method 1) based on Spearman rank correlation coefficients in 30 winter annual species. Only significant (p≤0.05) correlation coefficients are shown. Note that LDMC is the inverse of leaf water content (LWC in mg/g, LWC = 1000-LDMC).


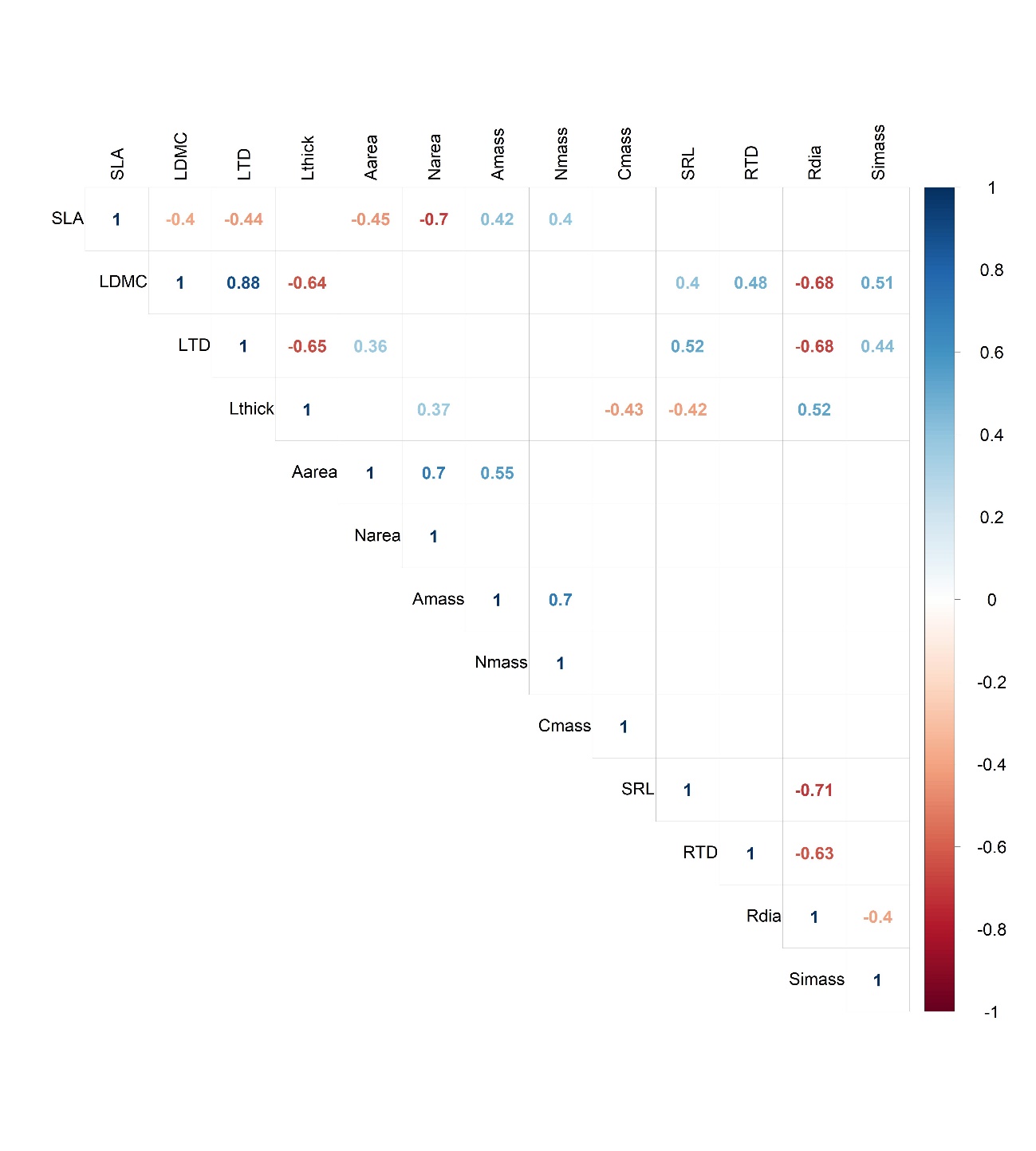


**Supplementary Table S5.** Relations between species’ relative growth rate and species’ scores along the main trait axes (PC 1, PC 2), or single traits in 19 winter annual species. Given are the results of linear models. Traits significantly related to relative growth rate are highlighted in bold; traits remaining significant after Holm-Bonferroni correction for 14 tests (traits) are indicated with *. For trait abbreviations see Table 1.

| Trait | F _1,17_ | p | R² |
| --- | --- | --- | --- |
| PC 1 (structural trait axis) | 0.08 | 0.786 | -0.05 |
| **PC 2 (carbon gain trait axis)** | **5.95** | **0.026** | **0.22** |
| SLA | 0.57 | 0.461 | -0.02 |
| LDMC | 0.07 | 0.792 | -0.05 |
| LTD | 0.01 | 0.910 | -0.06 |
| Lthick | 0.70 | 0.416 | -0.02 |
| **A**_area_**^a^** | **4.61** | **0.046** | **0.17** |
| N_area_ | 0.18 | 0.673 | -0.05 |
| **A**_mass_***^a^** | **15.19** | **0.001** | **0.44** |
| N_mass_ | 2.34 | 0.144 | 0.07 |
| C_mass_^a^ | 1.46 | 0.243 | 0.03 |
| SRL | 0.17 | 0.687 | -0.05 |
| RTD | 0.09 | 0.770 | -0.05 |
| Rdia | 0.15 | 0.707 | -0.05 |

^a^ were influenced by *Torilis tenella*, the species with by far the lowest relative growth rate; without *Torilis tenella*: A_area_ F_1,16_=2.78, p=0.115, R²=0.09; A_mass_ F_1,16_=10.77, p=0.005, R²=0.36; C_mass_ F_1,16_=4.16, p=0.058, R²=0.16

**Supplementary Table S6.** Relations between species’ mean annual rainfall niche (i.e. species’ distribution along the rainfall gradient) and species’ scores along the main trait axes (PC 1, PC 2), relative growth rate, or single traits. Given are the results of linear models. Traits significantly related to mean annual rainfall niche are highlighted in bold; traits remaining significant after Holm-Bonferroni correction for 15 tests (traits) are indicated with *. All traits were measured in 30 species (F_1,28_) except of relative growth rate measured in 19 species (F_1,17_). For trait abbreviations see Table 1.

| Trait | F | p | R² |
| --- | --- | --- | --- |
| PC 1 (structural trait axis) | 2.25 | 0.145 | 0.04 |
| PC 2 (carbon gain trait axis) | 0.50 | 0.486 | -0.02 |
| RGR | 0.31 | 0.587 | -0.04 |
| SLA | 0.63 | 0.434 | -0.01 |
| LDMC | 0.56 | 0.460 | -0.02 |
| LTD | 0.65 | 0.426 | -0.01 |
| Lthick | 0.89 | 0.353 | 0.0 |
| A_area_ | 0.04 | 0.837 | -0.03 |
| N_area_ | 0.02 | 0.882 | -0.03 |
| A_mass_ | 0.00 | 0.967 | -0.04 |
| N_mass_ | 0.69 | 0.413 | -0.01 |
| C_mass_ | 0.12 | 0.734 | -0.03 |
| SRL | 0.01 | 0.940 | -0.04 |
| **RTD*** | 17.96 | <0.001 | 0.37 |
| **Rdia** | 4.48 | 0.043 | 0.11 |

**Supplementary Method 1**. Measurement of leaf silicon content and turgor loss point.

Both leaf silicon content (Si_mass_) and turgor loss point were assessed in all 30 investigated annuals on 6-11 individuals per species.

Leaf silicon content (Si_mass_) was determined on the total leaf biomass harvested at the end of the plant’s life cycle. Silicon was extracted by an alkaline method using 30 mg of oven-dried, grounded leaf material (bulk sample) and 30 ml of 0.1 M sodium carbonate solution (Na_2_CO_3_) in a water bath for five hours (see Struyf et al., 2010). The solution was subsequently passed through a 0.2 µm syringe filter (ChromafilXtra CA-20/25) and its silicon concentration was determined with inductively coupled plasma optical emission spectrometry (ICP-OES) using a Varian Vista-Pro Radial element analyser (Varian Inc., Palo Alto, USA).

Turgor loss point was calculated based on osmotic potential at full turgor determined with an osmometer (Vapor Model 5600, Wescor, Logan, Utah, USA) on leaf discs (one disc per plant) from plants fully hydrated overnight 8-12 weeks after sowing following Bartlett et al. (2012) and Sun et al. (2020).

**References**

Bartlett, M. K., Scoffoni, C., Ardy, R., Zhang, Y., Sun, S., Cao, K., et al., (2012). Rapid determination of comparative drought tolerance traits: using an osmometer to predict turgor loss point. *Methods Ecol. Evol.* 3, 880–888. doi: 10.1111/j.2041-210X.2012.00230.x

BioGIS. (2018). Israel Biodiversity Information System. <http://www.biogis.huji.ac.il>. [Accessed October 30, 2018]

Kattge, J., Díaz, S., Tautenhahn, S., Werner, G. D. A., Aakala, T., Abedi, M., et al. (2020). TRY plant trait database–enhanced coverage and open access. *Glob. Change Biol*. 26, 119–188. doi: 10.1111/gcb.14904

Struyf, E., Mörth, C.‐M. C. Humborg, C., and Conley D. J. (2010). An enormous amorphous silica stock in boreal wetlands, *J. Geophys. Res*. 115, G04008. doi:10.1029/2010JG001324

Sun, S., Jung, E., Gaviria, J., and Engelbrecht, B. M. J. (2020). Drought survival is positively associated to high turgor loss points in temperate perennial grassland species. *Funct. Ecol.* 34, 788–798. doi: 10.1111/1365-2435.13522
